# A multidimensional landscape of extracellular vesicle-mediated tumor drug resistance: mechanisms, biomarkers, engineered drug delivery, and clinical translation

**DOI:** 10.20517/evcna.2025.184

**Published:** 2026-05-07

**Authors:** Zhiqin Fu, Yanting Kuang, Meichai Li, Kexuan Zhou, Hao Shi, Yongwei Gu, Xin Wu, Yizhun Zhu, Jiyong Liu

**Affiliations:** ^1^School of Pharmacy, Faculty of Medicine, Macau University of Science and Technology, Macau SAR, China.; ^2^Department of Pharmacy, Huashan Hospital, Fudan University, Shanghai 200040, China.; ^3^Shanghai WeiEr Lab, Shanghai 201707, China.; ^4^Department of Pharmacy, Fudan University Shanghai Cancer Center, Shanghai 200032, China.; ^5^Department of Oncology, Shanghai Medical College, Fudan University, Shanghai 200032, China.; ^#^These authors contributed equally to this work.

**Keywords:** Extracellular vesicles, cancer therapy, drug resistance, biomarkers, drug delivery, clinical application

## Abstract

Tumor drug resistance is a major clinical challenge that limits the efficacy of chemotherapy, targeted therapy, and immunotherapy, thereby contributing to tumor recurrence, metastasis, and reduced overall patient survival rates. Recent studies reveal that extracellular vesicles (EVs) in the tumor microenvironment act as key mediators of intercellular communication. They play a central role in mediating tumor cell resistance by transporting functional cargo, including RNA, proteins, and lipids. This review outlines the mechanisms of EV-mediated tumor resistance, including key processes such as drug efflux, evasion of apoptosis, maintenance of epithelial-mesenchymal transition and cancer stem cell phenotypes, remodeling of the immune microenvironment, metabolic reprogramming, and expansion of resistant cell populations. It also discusses the use of EVs as biomarkers of resistance and their associated detection technologies. Furthermore, this paper highlights therapeutic strategies for reversing drug resistance through engineered EVs, including the delivery of small molecules, nucleic acid therapeutics, and key bioactive components. It also reviews current preclinical studies and progress toward clinical translation of EV-based resistance reversal strategies. This review aims to elucidate the role and translational potential of EVs in tumor drug resistance through a systematic approach that integrates mechanism exploration, biomarker identification, engineered drug delivery, and clinical translation. It provides a comprehensive reference to facilitate further advances in this field, from basic research to clinical practice.

## INTRODUCTION

Tumor drug resistance represents one of the primary challenges in contemporary cancer treatment. Whether in traditional chemotherapy, precision-targeted therapy, or emerging immunotherapy, the development of resistance significantly diminishes clinical efficacy, becoming a key driver of treatment failure, tumor recurrence, and metastasis^[1,2]^. Traditional research has predominantly focused on resistance mechanisms within tumor cells themselves, such as genetic mutations, activation of drug efflux pumps, or compensatory activation of signaling pathways. However, mounting evidence indicates that resistant tumor cells not only enhance their own resistance but also spread this resistance to neighboring sensitive cells through complex microenvironmental signaling networks. This ultimately leads to worsened patient prognosis and significantly reduced survival rates^[3]^. Therefore, elucidating the fundamental mechanisms underlying drug resistance and metastasis, identifying early warning indicators, and developing novel strategies to effectively reverse or overcome resistance have become critical components in current oncology research and clinical translation.

In recent years, extracellular vesicles (EVs) have rapidly emerged as a focal point of research among oncologists due to their pivotal role in drug resistance formation and metastasis within the tumor microenvironment. EVs are actively secreted by cells as lipid bilayer vesicles measuring approximately 30-150 nanometers in diameter^[4,5]^. Serving as vital signaling carriers for intercellular communication, they load and transport diverse functional molecules, such as proteins, nucleic acids, lipids, and metabolites, throughout the tumor microenvironment^[6-8]^. Drug-resistant tumor cells release EVs to deliver specific resistance-associated cargo, including drug efflux pump proteins, pro-survival signaling proteins, and resistance-related noncoding RNAs (ncRNAs), to drug-sensitive recipient cells. This process rapidly propagates the resistance phenotype throughout the cell population, establishing a multidimensional, dynamic resistance network^[9]^. EVs serve not only as carriers of information but also as pivotal hubs connecting tumor cells with their microenvironment, coordinating collective drug resistance responses^[10]^. Therefore, elucidating the specific mechanisms underlying EV formation and the transfer of drug resistance, while exploring novel strategies to effectively reverse resistance, represents a critical focus in current cancer therapy.

The emergence and reversal of cancer drug resistance are multistep, dynamically evolving systemic processes. However, most existing reviews focus on a single aspect, such as examining the functional role of a single cargo molecule [e.g., a specific microRNA (miRNA)] within EVs in drug resistance, or on their engineered application as drug delivery vehicles. Recent literature lacks a comprehensive, integrated perspective that bridges “mechanism exploration - biomarker identification - engineered drug delivery - clinical translation”. To address this gap, this paper aims to provide an innovative, systematic, and holistic review. We focus on an in-depth discussion across four interrelated dimensions: (1) Systematically map the core molecular mechanisms of EV-mediated tumor drug resistance, encompassing key pathways such as drug efflux, evasion of apoptosis, epithelial-mesenchymal transition (EMT) and maintenance of cancer stem cell (CSC) phenotype, remodeling of the immune microenvironment, metabolic reprogramming, and proliferation of drug-resistant populations; (2) Provide a detailed review of recent advances and challenges in utilizing EVs as biomarkers for drug resistance, including EV detection technologies and their translational applications; (3) Present a focused discussion on therapeutic strategies involving engineered EVs to reverse drug resistance, including their use as targeted drug delivery vehicles synergizing with small molecules, nucleic acids, and Traditional Chinese medicine (TCM), as well as cutting-edge designs combining nanomaterials to construct hybrid delivery systems; (4) Analyze current preclinical research and clinical translation progress in EV-based resistance reversal strategies, while outlining future translational pathways. Using this four-part framework, this paper systematically constructs a comprehensive map of EVs’ role in tumor drug resistance. It particularly emphasizes strategies for reversing drug resistance through engineered EVs, providing systematic references and insights to advance this field, from basic research to clinical practice.

## EV-MEDIATED MECHANISMS OF TUMOR DRUG RESISTANCE

**Figure 1:**
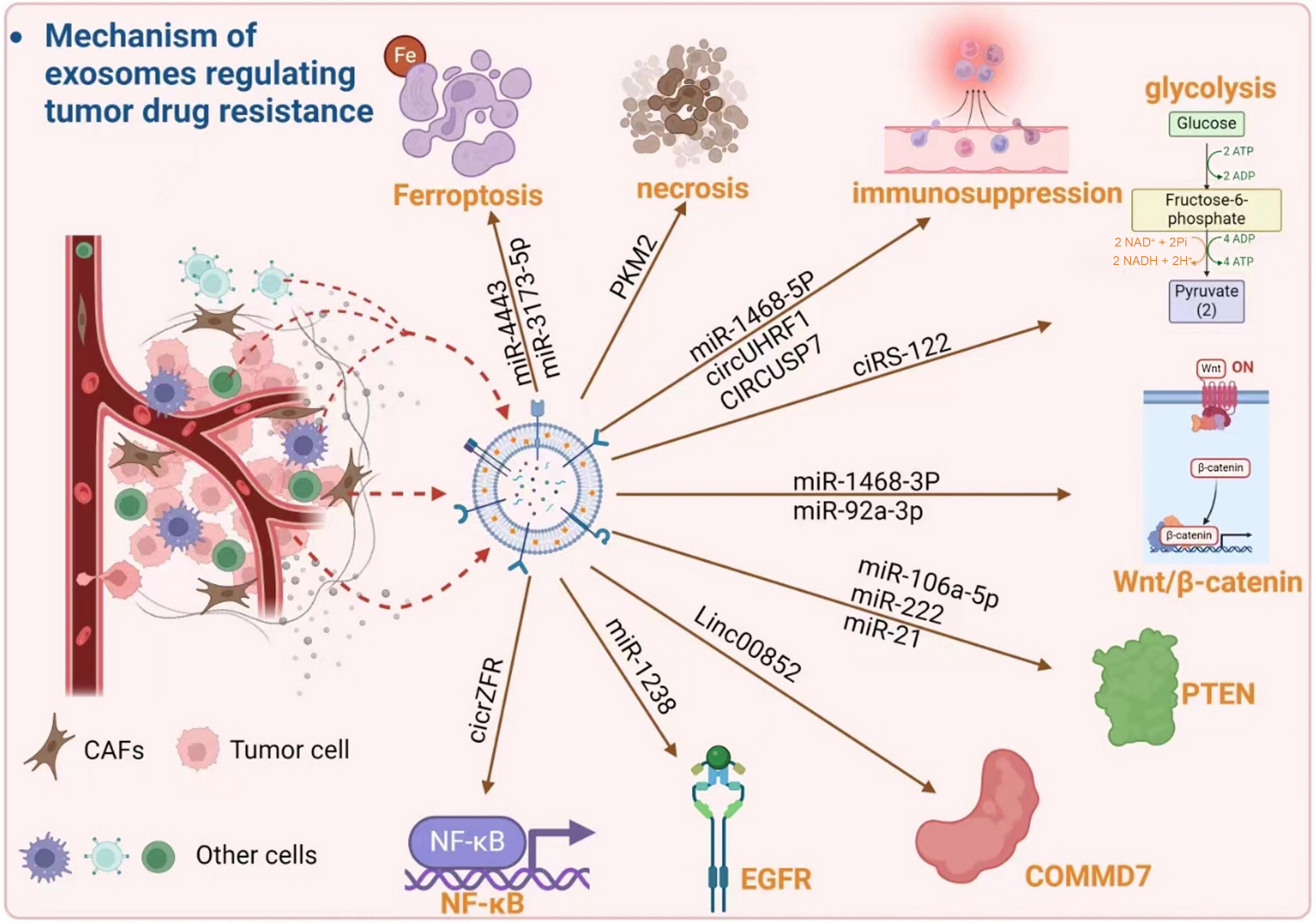
Mechanisms by which EVs regulate tumor drug resistance involving multiple factors and pathways^[12]^. EVs: Extracellular vesicles; CAFs: cancer-associated fibroblasts; miR: microRNA; circRNA: circular RNA; ciRS-122: circular RNA sponge for miR-122; circUHRF1: circular RNA UHRF1; CIRCUSP7: circular RNA USP7; Linc00852: long intergenic non-protein coding RNA 852; NF-κB: nuclear factor kappa-B; EGFR: epidermal growth factor receptor; PKM2: pyruvate kinase M2; PTEN: phosphatase and tensin homolog; Wnt: wingless-related integration site signaling pathway; β-catenin: beta-catenin; COMMD7: copper metabolism MURR1 domain-containing protein 7; ATP: adenosine triphosphate; ADP: adenosine diphosphate; NAD+: nicotinamide adenine dinucleotide (oxidized form); NADH: nicotinamide adenine dinucleotide (reduced form); Fe: iron.

The development of tumor drug resistance remains a major obstacle to therapeutic efficacy. Current evidence indicates that EVs play a pivotal role in the onset and progression of tumor drug resistance [Figure 1]^[11,12]^. The established mechanisms of EV-mediated tumor drug resistance primarily involve drug efflux, the inhibition of apoptotic pathways, the maintenance of EMT and CSC phenotypes, the remodeling of the immune microenvironment, the reprogramming of drug metabolism, and the proliferation of drug-resistant populations^[13]^.

### EVs promote drug efflux

Multidrug resistance (MDR) is associated with increased expression of adenosine triphosphate (ATP)-binding cassette (ABC) transporters. These transporters utilize energy from ATP hydrolysis to accelerate the efflux of anticancer drugs from target cells, preventing drug accumulation and thereby reducing therapeutic efficacy^[14]^. The multidrug resistance protein 1 gene (*MDR1*, *ABCB1*) encodes the key drug transporter P-glycoprotein (P-gp), which is expressed in over 50% of MDR-phenotypic tumors^[15]^. Multiple cytotoxic drugs, including paclitaxel (PTX) and doxorubicin (DOX), serve as substrates for P-gp.

Notably, EV-mediated dissemination of ABC transporters represents a key mechanism by which drug efflux-driven resistance can be transmitted between tumor cells, rather than remaining confined to intrinsically resistant populations. Extensive experimental evidence indicates that P-gp and other MDR transporters can be transferred from drug-resistant tumor cells to drug-sensitive tumor cells via circulating EVs, leading to acquired drug resistance in recipient cells^[16]^. Studies have shown that, in cancer cell models, DOX significantly increases the release of ABCB1-containing EVs from resistant cells. By downregulating Rab5 to accelerate EV circulation, this process markedly enhances intercellular ABCB1 transfer, thereby enabling sensitive cancer cells to develop an rapidly acquired resistance phenotype and evade the cytotoxicity of chemotherapeutic drugs^[17]^. Furthermore, elevated levels of Circ_0076305 in EVs derived from non-small cell lung cancer (NSCLC) resistant cells enhance ABCC1 [multidrug resistance protein 1 (MRP1)] expression by regulating miR-186-5p, thereby promoting cisplatin efflux in NSCLC and increasing cellular resistance^[18]^. *In vitro* studies using prostate cancer models have confirmed that MDR1/P-gp is transported via EVs to docetaxel-sensitive cells, leading to acquired docetaxel resistance^[19]^.

From a therapeutic perspective, EV-mediated drug efflux represents a highly actionable resistance mechanism, as both the cargo (e.g., ABCB1/ABCC1 and regulatory RNAs) and the intercellular transfer process itself are amenable to intervention. Accordingly, engineered EV-based strategies designed either to block transporter transfer or to deliver nucleic acid therapeutics targeting ABC transporters may offer a direct and efficient approach to reversing efflux-driven MDR.

### EVs modulate apoptosis and autophagy to promote drug resistance

In cancer chemotherapy, induction of apoptosis in cancer cells is a core mechanism of drug efficacy. However, tumor cells can attenuate apoptotic signaling through intercellular communication, thereby enabling survival under drug stress. Apoptosis resistance is thus a key mechanism of anticancer drug resistance. As crucial intercellular messengers, EVs promote cancer cell drug resistance by delivering anti-apoptotic factors and transporting regulatory molecules.

EVs carrying overexpressed tyrosine kinase with immunoglobulin-like and epidermal growth factor (EGF)-like domains 1 (TIE-1), when transferred to cisplatin-sensitive ovarian cancer cells, confer resistance to apoptosis by suppressing DNA damage responses, thereby promoting cisplatin resistance^[20]^. Elevated plasma gelsolin (pGSN) expression correlates with reduced overall survival and recurrence-free survival in ovarian cancer patients. pGSN secreted by EVs upregulates its own expression through the α5β1 integrin-focal adhesion kinase (FAK)-Akt (protein kinase B)-hypoxia-inducible factor (HIF) 1α signaling pathway, thereby inhibiting cisplatin-induced apoptosis in ovarian cancer cells and conferring cisplatin resistance to cells originally sensitive to chemotherapy^[21]^.

Studies investigating the effects of hypoxia-induced EVs in NSCLC have demonstrated that hypoxia exacerbates cisplatin resistance in lung cancer cells. Under hypoxic conditions, EVs secreted by cisplatin-resistant cells show increased pyruvate kinase M2 (PKM2) expression, which inhibits the ubiquitin-mediated degradation of Bcl-2, thereby suppressing apoptosis and conferring cisplatin resistance to sensitive NSCLC cells^[22]^. Au Yeung *et al*. also identified high levels of miR-21 in EVs secreted by cancer-associated adipocytes (CAAs) and cancer-associated fibroblasts (CAFs) from advanced ovarian cancer^[23]^. miR-21 transferred from CAAs and CAFs to ovarian cancer cells downregulated its target apoptotic protease-activating factor 1 (APAF1), thereby inhibiting apoptosis and enhancing drug resistance in ovarian cancer cells.

Collectively, EV-mediated modulation of apoptosis and autophagy primarily functions as a resistance-supporting mechanism rather than a dominant driver of drug resistance. From a therapeutic perspective, this pathway is conditionally actionable and is more likely to contribute to resistance maintenance or amplification, thereby often requiring combination strategies rather than serving as a standalone target for resistance reversal.

### EVs drive EMT and CSC maintenance

EMT refers to the biological process whereby epithelial cells, under specific signaling stimuli, lose their original polarity and intercellular junctions while acquiring the migratory, invasive, and anti-apoptotic properties of mesenchymal cells. In tumors, EMT is a key step that enables cancer cells to acquire local invasion and distant metastasis capabilities. CSCs represent a small subpopulation within tumors that possess self-renewal capacity, unlimited proliferative potential, and multipotent differentiation ability.

Within the context of drug resistance, EMT and CSC properties primarily contribute to phenotypic plasticity, allowing tumor cells to adapt dynamically to therapeutic stress rather than directly conferring resistance through a single dominant pathway. EVs act as crucial mediators within the EMT-CSC axis, endowing tumor cells with drug resistance and continuously driving tumors toward higher malignancy^[24]^.

**Figure 2:**
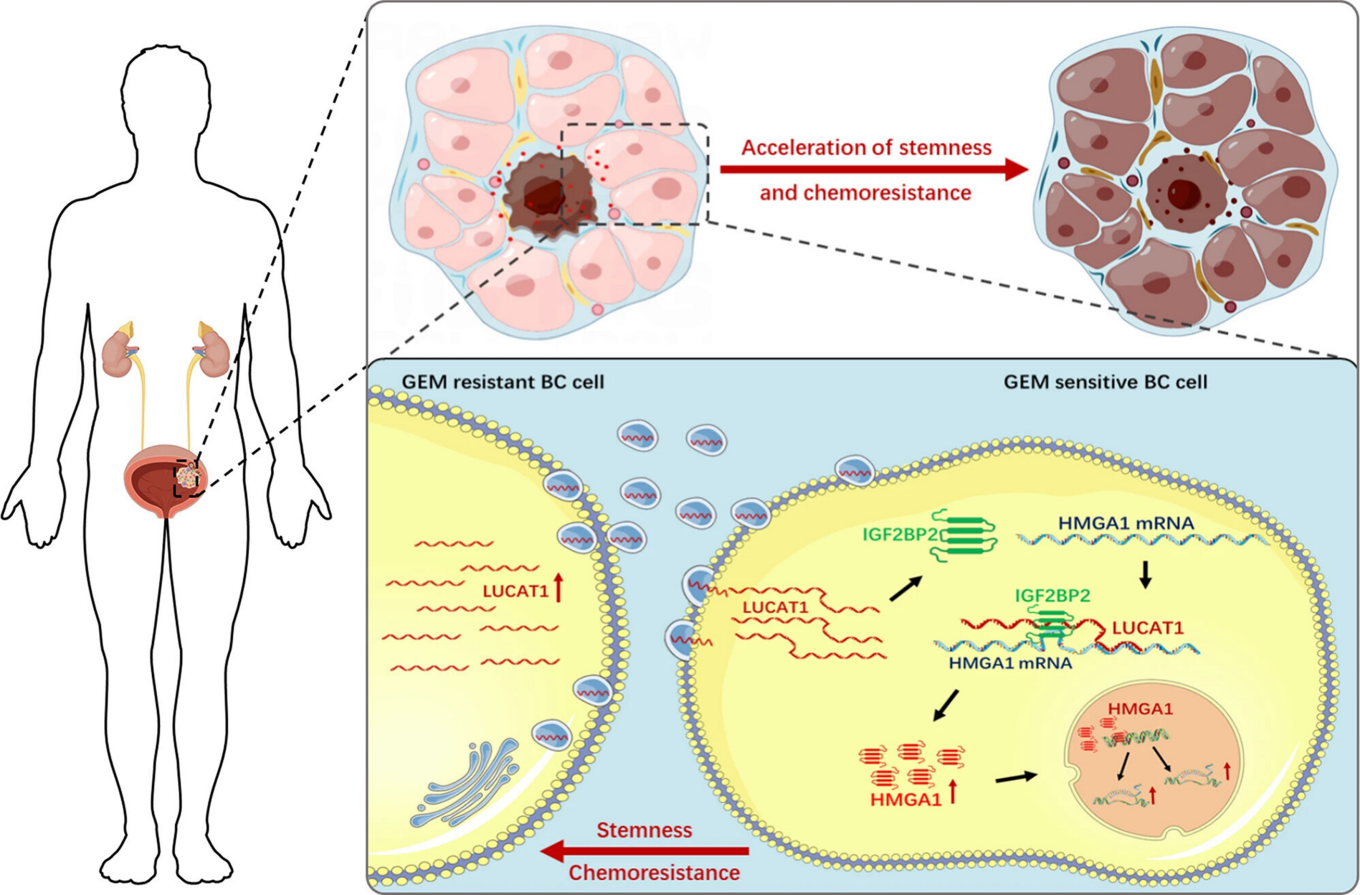
Schematic diagram of the oncogenic role of EV-transmitted LUCAT1 in bladder cancer. EV-transmitted LUCAT1 promotes the stemness phenotype and chemoresistance of BC cells via upregulating HMGA1 expression via binding to IGF2BP2, thus contributing to its oncogenic activity in bladder cancer pathogenesis^[28]^. EVs: Extracellular vesicles; BC: bladder cancer; GEM: gemcitabine; LUCAT1: lung cancer-associated transcript 1; HMGA1: high mobility group AT-hook 1; IGF2BP2: insulin-like growth factor 2 mRNA-binding protein 2; mRNA: messenger RNA.

CAFs can secrete miR-92a-3p-enriched EVs into the tumor microenvironment. miR-92a-3p promotes EMT in colorectal cancer (CRC) cells by targeting F-box and WD repeat domain-containing 7 (FBXW7) and modulator of apoptosis protein1 (MOAP1), thereby leading to resistance against fluorouracil and oxaliplatin (OXA)^[25]^. Other studies have reported that miR-155 upregulation in DOX- and PTX-resistant cells correlates with EMT. Co-culturing DOX- and PTX-sensitive cells with resistant cell-derived EVs elevates their miR-155 levels and induces chemotherapy resistance^[26]^. Shan *et al*. reported that EVs secreted by CAFs reduce apoptosis in bladder cancer cells by enhancing EMT, thereby promoting metastasis and chemotherapy resistance, as evidenced by increased N-cadherin and vimentin and decreased E-cadherin expression^[27]^. Furthermore, the lung cancer associated transcript 1 (LUCAT1) delivered by EVs can promote the CSC phenotype of bladder cancer [Figure 2]^[28]^.

Furthermore, chemotherapy induces breast cancer cells to secrete EVs carrying miR-9-5p, miR-195-5p, and miR-203a-3p. These miRNAs stimulate breast cancer cells to acquire a CSC phenotype, thereby conferring therapeutic resistance to tumor cells^[29]^.

From a therapeutic perspective, EV-driven EMT-CSC plasticity represents an important resistance amplifier rather than a directly targetable resistance determinant. Although disrupting this axis may enhance treatment efficacy, effective resistance reversal is more likely to require combination strategies that concurrently target dominant resistance drivers and EMT-CSC-associated adaptive programs.

### EVs remodel the immune microenvironment to induce immune therapy resistance

A significant challenge in current cancer treatment is the pronounced immune suppression observed in patients^[30]^. During tumor progression, EVs play a central role in shaping the immunosuppressive microenvironment and mediating immune escape. By carrying various immunosuppressive cargo, EVs suppress antitumor immune responses through multiple mechanisms and targets, thereby promoting tumor proliferation, metastasis, and resistance. Notably, EV-mediated immune modulation represents a dominant and clinically relevant mechanism underlying immunotherapy resistance, and EVs function as key drivers of immunotherapy failure^[31,32]^.

Researchers discovered that mouse mammary tumor EVs selectively reduce perforin release, block janus kinase 3 (Jak3) and cyclin D3 expression, and prevent natural killer (NK) cell entry into the cell cycle, thereby suppressing NK cell activity^[33]^. Related studies report that, compared to EVs from normoxic tumor cells, hypoxia-derived tumor cell EVs carry transforming growth factor (TGF)-β1 and miR-23a, which significantly inhibit NK cytotoxicity across multiple tumor models^[34]^. Research indicates that tumor cell-derived EVs can systematically downregulate natural killer group 2D (NKG2D) expression via NKG2D ligands and TGF-β1, thereby impairing lymphocytes’ ability to recognize and kill tumor cells^[35]^.

From a clinical and translational perspective, EV-mediated immune suppression constitutes a highly actionable resistance mechanism, particularly in the context of immune checkpoint blockade failure. Because EV-derived immunoregulatory cargo directly interferes with cytotoxic lymphocyte function and immune recognition, targeting EV-mediated immune modulation offers a rational and mechanistically aligned strategy for restoring antitumor immunity and overcoming immunotherapy resistance.

### EVs induce metabolic reprogramming to form drug-resistant adaptive states

During cancer progression, cancer cells exhibit high metabolic plasticity to adapt to dynamic changes in the tumor microenvironment^[36]^. A common metabolic feature of cancer cells is their ability to acquire essential nutrients from nutrient-depleted environments and utilize these nutrients to sustain survival, proliferation, and growth. Thus, metabolic reprogramming is considered a hallmark of cancer^[37]^.

Recent studies have demonstrated that EVs regulate processes such as glucose metabolism, lipid metabolism, and oxidative pathways in cancer cells by delivering cargo including proteins, metabolic enzymes, lipids, and nucleic acids, thereby enabling cancer cells to develop a drug-resistant adaptive state. One research team investigated the molecular mechanisms and biological roles of platelet-derived long noncoding RNAs (lncRNAs) in EV-mediated cancer progression within CRC^[38]^. Their findings revealed that, compared to healthy individuals, LINC00183 exhibited the most significant upregulation among platelet-derived EVs (PLT-Exos) in CRC patients^[38]^. They further demonstrated that LINC00183 interacts with enolase 1 (ENO1) to activate glycolysis in CRC cells, leading to lactate accumulation, H3K18 acetylation, and transcriptional upregulation of the oncogene growth differentiation factor (GDF) 15, thereby promoting tumor progression. Furthermore, drug-resistant triple-negative breast cancer (TNBC) cells upregulate the endogenous synthesis of lipids such as arachidonic acid (AA), leading to the accumulation of lipid droplets within cancer cells. Through the local release of AA-rich EVs, these cells regulate programmed cell death ligand 1 (PD-L1) overexpression and prostaglandin E2 (PGE2) production in neutrophils within the tumor microenvironment. This process inhibits CD8+ T cell infiltration into tumors and induces T cell exhaustion, thereby promoting TNBC resistance^[39]^. Yang *et al*. investigated the effects of glioblastoma-derived EVs (GDEs) on ferroptosis in dendritic cells (DCs)^[40]^. Their results showed that GDEs induce ferroptosis in DCs by elevating Fe^2+^ levels, reactive oxygen species (ROS), and lipid peroxidation while decreasing nuclear factor erythroid 2-related factor (NRF)2, SLC7A11, and glutathione peroxidase (GPX) 4 protein expression, thereby suppressing immune responses.

From a therapeutic standpoint, EV-mediated metabolic reprogramming represents a system-level adaptive mechanism that is strategically important yet challenging to target directly. Accordingly, interventions aimed at metabolic pathways are more likely to enhance treatment efficacy when combined with therapies targeting dominant resistance drivers, rather than serving as standalone strategies for resistance reversal.

### EV-mediated spread of drug-resistant populations

Given the heterogeneous drug sensitivity within tumors, EVs released by drug-resistant cells can be taken up by drug-sensitive populations, thereby facilitating the intercellular dissemination of resistance and contributing to tumor relapse and therapeutic failure. This process reflects a population-level resistance propagation mechanism rather than isolated intracellular events.

Studies have shown that oxaliplatin-resistant/5-fluorouracil-resistant (OXR/FUR) CRC cells transmit specific integrins (ITGβ3 and ITGαv) via EVs, conferring drug resistance and invasive potential to recipient cells^[41]^. In addition, tumor-derived EVs enriched in PD-L1 have been implicated in resistance to anti-PD-1 therapy^[42]^. Together, these findings indicate that resistance dissemination is actively coordinated through EV-mediated intercellular communication.

Accordingly, strategies targeting single pathways or individual tumor cells are often insufficient to prevent the spread of resistance. Effective anti-resistance interventions should instead account for EV-mediated signaling networks that integrate multiple resistance mechanisms at the multicellular level.

## EVS AS DRUG RESISTANCE BIOMARKERS

EVs not only play a vital role in material and information transfer within normal cells but are also closely associated with tumor development and progression, making them promising biomarkers for the early diagnosis of various cancers [Table 1]. When excreted from tumor cells, the substances carried by EVs closely resemble those found within the original secreting cells. Consequently, real-time monitoring of changes in EV content provides crucial evidence for precision medicine needs in diagnosis, prognosis, and disease surveillance. Compared to tissue biopsy, liquid biopsy offers distinct advantages in identifying tumor markers: minimal invasiveness, ease of acquisition, rapid testing, and cost-effectiveness^[50]^.

**Table 1:** The role of EV biomarkers in tumor resistance

**EV biomarkers**	**Type**	**Analytical approach**	**Mechanism**	**Drug**	**Cancer**	**Ref.**
LUCAT1	RNA	qRT-PCR	Enhance the phenotype of CSCs and promote drug resistance	GEM	Bladder cancer	[28]
miR-21-5p	RNA	Differential centrifugation	Metabolic reprogramming leads to drug resistance	Cisplatin	Ovarian cancer	[43]
miR-1246	RNA	Differential centrifugation	Enhance the phenotype of CSCs and promote drug resistance	Cisplatin	Lung cancer	[44]
Circ_FMN2	RNA	qPCR	Inhibit apoptosis of cancer cells and promote drug resistance	Anti-cancer drug	Colorectal cancer	[45]
Recombinant SLC1A5	Protein	Differential centrifugation	Metabolic reprogramming leads to drug resistance	TKI	NSCLC	[46]
PDL1	Protein	Ultracentrifugation	Inhibiting T cell activation leads to immune therapy resistance	ICI	MM	[47]
IDH1	Protein	Ultracentrifugation	Tumor metabolic reprogramming leads to the emergence of drug-resistant phenotypes.	5FU	Colorectal cancer	[48]
Heat shock protein (HSP40) homolog, subfamily B, member 8 (DNAJB8)	Protein	Density gradient centrifugation, quantitative reverse transcription-PCR (qRT-PCR) and Western blot analysis	Inhibits TP53 ubiquitination and degradation leading to MDR1 upregulation and promote drug excretion	Oxaliplatin	Colon cancer (COAD)	[49]

EV: Extracellular vesicle; CSCs: cancer stem cells; GEM: gemcitabine; qRT-PCR: quantitative real-time polymerase chain reaction; qPCR: quantitative polymerase chain reaction; PDL1: programmed cell death ligand 1; ICI: immune checkpoint inhibitors; TKI: tyrosine kinase inhibitor; NSCLC: non-small cell lung cancer; MM: metastatic melanoma; 5-FU: 5-fluorouracil; MDR1: multidrug resistance protein 1; SLC1A5: solute carrier family 1, member 5; 5FU: 5-fluorouracil; IDH1: isocitrate dehydrogenase 1; HSP40: heat shock protein.

### Nucleic acid-loaded EVs (miRNA/lncRNA/circRNA) as drug resistance biomarkers

In recent years, a growing body of research has demonstrated that EVs carrying ncRNAs hold significant potential as therapeutic targets for cancer and as novel biomarkers for diagnosis and prognosis. The abnormal expression of these ncRNAs within EVs plays a crucial role in driving cancer development and progression^[51,52]^. EV RNA sequencing (EV-RNA-seq) enables the rapid and efficient acquisition of comprehensive information, making it an ideal approach for disease diagnosis and prognosis^[53,54]^. Current research on ncRNAs in EVs primarily focuses on the crucial regulatory roles of miRNA, lncRNA, and circular RNA (circRNA).

EVs can carry miRNAs to transfer genetic material between cells, enabling communication between neighboring and distant cells^[55]^. Numerous studies indicate that extracellular miRNA represents a promising noninvasive approach for early lung cancer diagnosis, as it aids in identifying pathological subtypes of cancer and may improve patient prognosis and quality of life. For example, serum EV-derived miR-21 expression levels in hepatocellular carcinoma (HCC) patients are reported to be 5.57-fold higher than in healthy controls and positively correlate with tumor staging (*P* = 0.001)^[56]^. Furthermore, analytical studies indicate that elevated EV-derived miR-21 and miR-10b levels predict poorer prognosis, and their combined application more effectively distinguishes early-stage HCC from healthy individuals^[57]^. One research team discovered that miR-660 indirectly downregulates the *p53* gene by activating mouse double minute 2 (MDM2), thereby inhibiting lung cancer development^[58]^. Furthermore, related studies indicate that the miR-34 family acts as a direct transcriptional target of p53 and contributes to the suppression of lung cancer cell proliferation^[59]^.

EV-derived lncRNAs hold promise as diagnostic biomarkers for cancer^[60]^. Studies have confirmed that the EV-derived lncRNA H19 promotes resistance to erlotinib in patients with NSCLC. EV-derived H19 modulates autophagy related 7 (ATG7) expression by targeting miR-615-3p, thereby influencing NSCLC cell resistance to erlotinib^[61]^. Furthermore, lncRNA metastasis associated lung adenocarcinoma transcript (MALAT)-1 is highly expressed in EVs isolated from the serum of NSCLC patients, and EV MALAT-1 levels correlate positively with tumor-node-metastasis (TNM) staging and lymph node metastasis^[62]^.

Some circRNAs have also been identified as potential novel biomarkers and targets for cancer diagnosis and treatment^[63]^. Hsa_circ_0010235 (circ_0010235) is a circRNA derived from antisense splicing of exon A1 of aldehyde dehydrogenase 4 family member A1. Zhu *et al*. found that circ_0010235 modulates homeobox A10 (HOXA10) expression by binding miR-588, thereby enhancing radiation resistance in NSCLC^[64]^. Furthermore, circ_0010235 accelerates immune escape in HCC cells by suppressing miR-636 and upregulating PD-L1 expression^[65]^.

EV-derived RNA is more stable than free RNA because the lipid bilayer structure of EVs protects RNA from degradation. When exomiR-99b-5p and exomiR-150-5p were mixed with RNase A, their expression levels showed no significant change. Similarly, after pretreatment with proteinase K and ribonuclease A, miRNAs extracted from the EV-enriched fraction (e.g., let-7b-3p, miR-150-3p, miR-145-3p, and miR-139-3p) showed only a slight decrease (approximately 10%), whereas miRNAs from the plasma fraction decreased by approximately 66% in most cases. EVs derived from cancer cells are widely present in the circulation due to the high metabolic activity of tumors, making them readily accessible for collection from the circulatory system^[66]^.

### EV-derived PD-L1 and immune-related proteins as markers of immune resistance

PD-1 is expressed on various immune cells, including peripherally activated T cells, B cells, and monocytes. PD-L1, a prototypical immune surface protein and the first identified PD-1 ligand, binds to PD-1 on the T cell plasma membrane. Recent studies indicate that EV-derived PD-L1 plays a crucial role in tumor immune suppression^[67-69]^. EV-derived PD-L1 inhibits T cell activity and enhances tumor cell immune tolerance, thereby blocking immune responses that could destroy tumors and leading to tumor immune escape. Extensive studies demonstrate abnormal PD-L1 expression across multiple tumor types, including skin, brain, thyroid, esophageal, and CRC. Consequently, PD-L1 is regarded as a key regulator of tumor immune escape. Research indicates that tumor cells weaken antitumor immunity by expressing biologically active PD-L1 on the surface of secreted EVs^[57,70]^.

EV-associated PD-L1 has been identified as a potential biomarker for melanoma. Compared with soluble PD-L1, EV-associated PD-L1 is less susceptible to degradation by extracellular proteases and can induce T cell dysfunction, thereby promoting tumor progression^[71]^. Further studies confirm that significantly elevated EV-associated PD-L1 correlates with tumor progression. In melanoma patients, circulating EV PD-L1 acts via T lymphocytes in secondary lymphoid organs and through the immunosuppressive PD-1/PD-L1 pathway^[72]^. The research team confirmed the presence of melanoma-associated EV PD-L1 and its immunosuppressive effects, proposing that EV PD-L1 levels could serve as a marker to distinguish clinical responders from non-responders. In metastatic melanoma patients, circulating EV PD-L1 levels positively correlate with interferon-gamma (IFN-γ) and change dynamically during anti-PD-1 therapy. The magnitude of early treatment-induced increases in circulating EV PD-L1, often regarded as an indicator of tumor cells’ adaptive response to T cell regeneration, distinguishes clinical responders from non-responders^[68]^. Studies reveal elevated TGF-β1 in EVs from advanced breast cancer drug-resistant cells. TGF-β1-mediated intercellular transfer via EVs enhances cell survival during drug treatment by inhibiting apoptosis and promoting cell viability through increased Smad2 phosphorylation^[73]^.

Proteomic analysis of EVs revealed the presence of multiple ABC transporters, including ABCB1 (MDR1/P-gp), ABCC1 (MRP1), ABCC10 (MRP7), and ABCG2 [breast cancer resistance protein (BCRP)/mitoxantrone resistance (MXR)], all of which are extensively associated with drug resistance^[74,75]^. Notably, EVs derived from ABCB1-expressing prostate and lung cancer cells mediate resistance to PTX and docetaxel, respectively^[76]^. Breast cancer cell-derived EVs mediate PTX resistance via ABCB2^[77]^, while ABCG2-encoding EVs enhance the transport of PTX, DOX, and topotecan^[78]^.

### Metalloproteinases as markers of immune resistance

EV-associated metalloproteinases have emerged as critical regulators of tumor progression and microenvironmental remodeling, yet they remain insufficiently discussed in the context of EV-mediated drug resistance. Recent evidence indicates that membrane-type matrix metalloproteinases [e.g., membrane type 1 matrix metalloproteinase (MT1-MMP)/MMP-14] and members of the a disintegrin and metalloproteinase (ADAM) family, such as ADAM10, are selectively incorporated into EVs and can retain proteolytic activity in the extracellular milieu. These vesicle-associated proteases contribute to extracellular matrix (ECM) degradation, thereby facilitating tumor invasion and metastatic dissemination. Notably, recent work has demonstrated that MT1-MMP can be trafficked into intraluminal vesicles and released via EVs, where it actively participates in pericellular ECM degradation and promotes invasive behavior^[79]^. Importantly, recent clinical studies have also highlighted ADAM10 and related metalloproteinases as potential biomarkers in cancer, underscoring their translational relevance^[80]^. Collectively, these findings position EV-associated metalloproteinases as key mediators linking vesicle trafficking with ECM dynamics, tumor microenvironment remodeling, and therapeutic resistance, highlighting their potential as both biomarkers and therapeutic targets.

### EV-derived lipids and metabolites as emerging resistance biomarkers

Research on EV-derived lipids as cancer biomarkers represents an emerging field^[81]^. Therefore, lipidomic analysis of circulating EVs can reflect cancer cell membrane characteristics and metabolic states. In a study examining drug-sensitive *vs*. drug-resistant NSCLC, researchers employed Matrix-assisted laser desorption ionization-time of flight mass spectrometry (MALDI-TOF-MS) to demonstrate that lipidomic signatures could distinguish gefitinib-sensitive cells from gefitinib-resistant cells based on EV composition. They detected 27 lipid features that were increased in resistant cells and 40 that were reduced in drug-sensitive cells. These lipids were identified as phosphatidylcholine and its ether-linked forms, lysophosphatidylcholine, sphingomyelins (SMs), phosphatidylglycerols (PGs), phosphatidylinositols (PIs), and lysophosphatidylinositols with different fatty acid residues^[82]^.

EV-derived metabolites can noninvasively and dynamically capture the overall metabolic adaptation state of tumors, offering a highly promising strategy for distinguishing treatment-sensitive from drug-resistant phenotypes, predicting therapeutic efficacy, and detecting drug resistance at an early stage. Researchers have examined EV biomarkers in acute myeloid leukemia (AML). Metabolomic studies indicate elevated levels of α-ketoglutarate in AML EVs. As a key intermediate in the Tricarboxylic Acid (TCA) Cycle and a precursor to glutamine, α-ketoglutarate functions as an antioxidant in multiple cellular processes and is critical for mitochondrial metabolism. Concurrently, glutathione peroxidase 3 (GPX3) is enriched in EVs. These molecules can be internalized by recipient cells, where they upregulate glutathione (GSH) levels and mitochondrial function while reducing ROS levels^[83]^.

### Functional phenotypes of EVs as biomarkers of drug resistance

Beyond the aforementioned traditional single-molecule biomarkers of EVs, their functional phenotypes can also serve as biomarkers of drug resistance^[84-86]^. Studies indicate that upregulation of miR-301a-3p, miR-21-5p, miR-106b, CKLF-like MARVEL transmembrane domain-containing 6 (CMTM6), and Tim-3, can induce M2 polarization of macrophages and significantly promote tumor cell migration, invasion, and EMT^[87-91]^. Conversely, elevated expression of miR-130 and miR-33 in EVs can exert antitumor effects by inducing M1 polarization of macrophages^[92,93]^. EVs derived from Tregs exhibit potent immunosuppressive effects, mediated by higher surface expression of factors such as miR-150-5p, miR-142-3p, CD25, cytotoxic T-lymphocyte-associated protein 4 (CTLA-4), and CD73^[94-96]^. Conversely, EVs from CD8+ T cells can directly inhibit tumor progression by regulating miR-765^[97]^. EVs derived from tumor cells carry miR-135b and miR-210, which suppress NK cell cytotoxic activity^[98,99]^. Conversely, EVs expressing NKG2D ligands, HSP70, miR-574, and miR-21 can bind to NK cells, thereby inducing their activation^[100,101]^. The functional phenotype of EVs, serving as a resistance biomarker, helps overcome the limitations of single-molecule markers by reflecting the dynamic, multitarget, and comprehensive effects of drug resistance.

### Clinical translation of EV detection technologies and liquid biopsy

**Figure 3:**
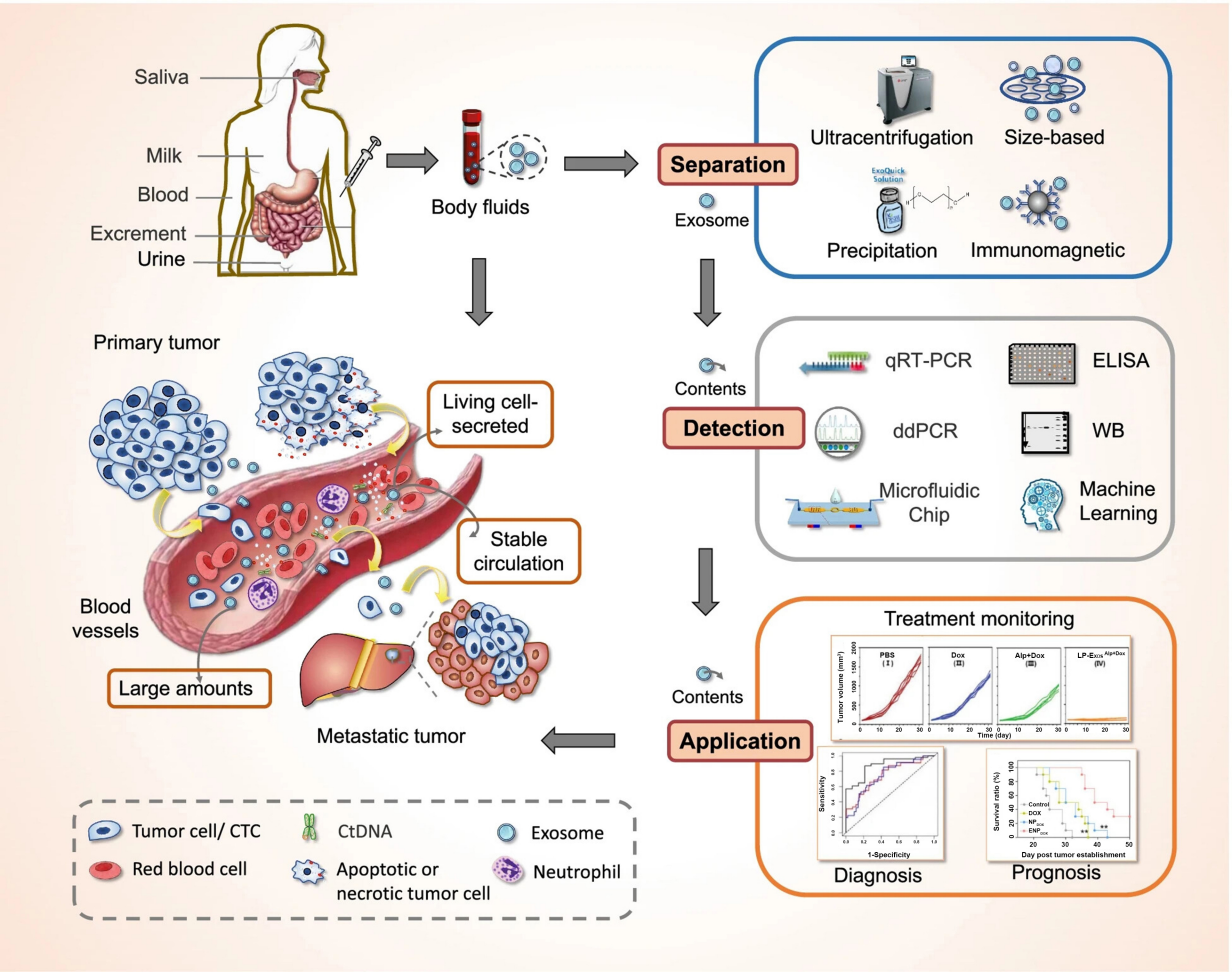
EVs as a new target for liquid biopsy. EVs are enriched in body fluids and are critically involved in tumorigenesis, tumor progression and metastasis^[104]^. EVs: Extracellular vesicles; CTC: circulating tumor cell; ctDNA: circulating tumor DNA; qRT-PCR: quantitative reverse transcription polymerase chain reaction; ddPCR: droplet digital polymerase chain reaction; ELISA: enzyme-linked immunosorbent assay; WB: Western blot.

As an ideal target for liquid biopsy in tumor drug resistance, EVs hold immense clinical potential, driving rapid advancements in detection technologies. However, the core challenge in transitioning from basic research to routine clinical testing lies in efficiently, specifically, and stably isolating EVs from complex biological fluids while performing highly sensitive quantitative analyses of their drug resistance-associated markers^[102]^. EV extraction forms the foundation for both research and clinical application. The entire process aims to efficiently and specifically isolate high-purity EVs from complex biological fluids such as plasma and cell culture supernatants. When selecting separation techniques, multiple factors must be comprehensively considered, including accessibility, yield, cost, required equipment, processing time, purity, functionality, and the intended application of purified EVs. Based on the biological, physical, and chemical properties of EVs, various separation and purification methods have been developed, playing a crucial supporting role in the clinical implementation of EV-based drug resistance markers. These include ultracentrifugation, ultrafiltration, size-exclusion chromatography (SEC), and immunocapture methods, among others [Figure 3]^[103,104]^.

Currently, ultracentrifugation is widely used for EV isolation due to its low cost and ease of operation. However, this method has certain limitations, including low yield and limited separation purity. Compared with ultracentrifugation, ultrafiltration offers faster processing and higher purity for EV separation, but it requires careful consideration of membrane specifications and carries a higher risk of sample loss. SEC offers high separation purity and efficiency but cannot effectively distinguish between vesicles of similar sizes. Immunocapture enables high-purity, specific targeting of EVs, yet it is limited by high costs, reliance on specific ligands, and low throughput^[105,106]^.

In addition to the commonly used EV isolation methods mentioned above, highly sensitive detection platforms are also available. EV separation systems based on microfluidic chips and nanoplasmonic sensor technology have emerged as widely used approaches for isolating EVs. This technology enables high-speed, high-throughput, highly precise, and cost-effective EV separation^[107,108]^. Digital droplet PCR (ddPCR) is an absolute quantitative method for nucleic acid detection that offers high sensitivity^[109]^, and EV-RNA-seq enables comprehensive analysis of RNA species and sequences within EVs^[110]^.

Although some of the aforementioned methods are considered cutting-edge technologies for EV isolation and characterization, many approaches still exhibit inherent limitations. Issues surrounding standardization and inter-batch comparability in critical stages - such as sample pretreatment, EV separation, detection, and data analysis - continue to hinder the widespread adoption of these techniques in clinical applications.

### Challenges and strategies for the clinical translation of EV biomarkers

Despite the promising roles of EV biomarkers in tumor drug resistance, their clinical translation remains hindered by several critical challenges, including limited specificity, lack of methodological standardization, and difficulties in dynamic monitoring^[111]^.

Limited specificity and pronounced heterogeneity represent major obstacles. Although tumor cells actively release EVs, these vesicles coexist with a substantial proportion of EVs derived from non-malignant cells in biological fluids, thereby reducing the relative abundance and detectability of tumor-associated signals. In addition, EVs do not constitute a homogeneous population but rather encompass a spectrum of vesicle subtypes with distinct biogenesis pathways, sizes, and molecular compositions. Meanwhile, EV cargo exhibits pronounced heterogeneity, influenced by factors such as tumor subtype, disease stage, and microenvironmental conditions, thereby complicating the identification of consistent and disease-specific biomarkers. To address these challenges, strategies including enrichment of tumor-derived EV subpopulations using specific surface markers, development of multi-analyte biomarker panels, and integration of multi-omics approaches (e.g., proteomics, transcriptomics, and lipidomics) are being actively explored to enhance diagnostic specificity and robustness^[112]^.

A lack of standardization across pre-analytical and analytical procedures further limits reproducibility and comparability between studies. Variations in sample collection, storage conditions, EV isolation methods (e.g., ultracentrifugation, SEC, immunoaffinity capture), and downstream characterization techniques can lead to inconsistent results. The establishment of standardized protocols and adherence to guidelines such as Minimal Information for Studies of Extracellular Vesicles (MISEV) are essential steps toward harmonizing EV-related methodologies. In addition, the development of robust, scalable, and clinically compatible isolation technologies is critical for routine clinical implementation^[113]^.

Challenges in dynamic monitoring also impede clinical application of EV biomarkers. Although EV biomarkers hold great potential for real-time monitoring of tumor evolution and therapeutic response, current detection methods are often time-consuming, labor-intensive, and lack sufficient sensitivity for longitudinal analysis. Furthermore, the temporal variability of EV release and cargo composition adds another layer of complexity. Emerging technologies, including microfluidic platforms, high-sensitivity biosensors, and single-EV analysis techniques, offer promising solutions for rapid, minimally invasive, and longitudinal monitoring^[114]^.

In summary, overcoming these challenges will require coordinated interdisciplinary efforts that integrate advances in bioengineering, molecular biology, and clinical research. The development of standardized workflows, highly specific biomarker panels, and sensitive detection platforms will be key to unlocking the full potential of EV biomarkers in precision oncology.

## ENGINEERED EVS AS A STRATEGY TO REVERSE DRUG RESISTANCE

In tumor drug resistance, EVs play a critical dual role. On one hand, they serve as carriers for intercellular communication, directly participating in the formation and spread of drug-resistant phenotypes. On the other hand, their excellent biocompatibility, low immunogenicity, natural membrane-crossing capabilities, and tissue tropism make them a highly promising delivery system for combating drug resistance. Following in-depth investigations into the complex origins of MDR, transforming EVs from messengers of resistance into tools to counteract resistance has emerged as a critical research direction for overcoming therapeutic bottlenecks in cancer treatment. In recent years, functional modifications of natural EVs through strategies such as genetic engineering, chemical modification, and nanomaterial hybridization have yielded engineered platforms capable of precise delivery of therapeutic molecules and actively overcoming resistance barriers. This section systematically reviews the construction strategies, mechanisms of action, and reversal potential of these engineered EVs across diverse resistance models, providing a comprehensive reference for developing next-generation targeted anti-resistance therapeutics.

### Engineering strategies for EV construction

Engineered EVs serve as precision modification platforms for natural nanocarriers, with their core construction centered on efficiently loading therapeutic molecules and conferring defined targeted functions. Based on intervention timing, existing technical systems are primarily categorized into endogenous loading and exogenous loading, which complement each other in underlying principles, advantages, and applicable scenarios.

Endogenous loading achieves preintegration of therapeutic molecules through genetic engineering at the donor cell level. This technology typically employs viral or non-viral vectors to introduce target sequences into cells, leveraging the natural EV biogenesis mechanism for encapsulation^[115]^. For instance, by overexpressing miR-218-5p in regulatory T cells, secreted EVs can target this molecule to podocytes in diabetic nephropathy, activating mitochondrial autophagy and mitigating damage^[116]^. Fusion protein-based strategies are equally effective. Cheng *et al*. expressed fusion proteins of Anti-CD3, Anti-EGFR (epidermal growth factor receptor), and PDGFR (platelet-derived growth factor receptor) on the EV membrane by transfecting Expi293F cells. This engineered EV population exhibited affinity for both breast cancer cells and T cells, enabling specific T cell enrichment near breast cancer cells to enhance their targeting and killing capacity^[117]^. Another team utilized a nanosecond pulse microfluidic system to prepare engineered EVs surface-modified with CD47, simultaneously overexpressing microRNA-29b to target hepatic stellate cells for treating liver fibrosis. This finding demonstrated that endogenous loading can concurrently achieve drug encapsulation and functional modification^[118]^. Endogenous loading exhibits high efficiency and approximates physiological conditions, with molecules protected by the lipid bilayer during encapsulation, thereby avoiding membrane structural damage from exogenous manipulation. This strategy is particularly prominent in nucleic acid drug delivery, especially suited for regenerative medicine scenarios requiring long-term, stable expression of miRNA or messenger RNA (mRNA). Its drawbacks include the lengthy development timelines to generate stably expressing engineered cell lines and limited compatibility with chemically synthesized drugs.

Exogenous loading refers to the process of introducing drug molecules into naturally isolated and purified EVs via various physical or chemical means. Common exogenous loading methods include electroporation, ultrasonication, extrusion, freeze-thaw cycles, and simple incubation. Electroporation creates transient microporous channels in the membrane via high-voltage pulses, offering advantages of precise quantification, strong controllability, and high reproducibility. However, electrostatic interactions may cause payload precipitation, and improper voltage control can damage EV structure^[119]^. Ultrasonication utilizes cavitation effects to facilitate transmembrane drug transport, exhibiting less EV aggregation than electroporation and relatively higher cellular uptake rates. Nevertheless, ultrasonic conditions may compromise EV structural integrity, and varying separation parameters can induce vesicle size and structural heterogeneity^[120]^. The extrusion method forces EVs through nanoscale membrane pores using mechanical pressure, offering simplicity but carrying a risk of vesicle rupture^[121]^. Although freeze-thaw methods yield stable results, their loading efficiency is lower than that of ultrasonic incubation, and they alter vesicle stability while reducing cellular uptake rates^[122]^. Simple incubation relies on concentration gradients to drive passive drug diffusion into EVs. Its advantage lies in the relative stability of chemical structures, but its drawbacks include low loading efficiency and limited control over drug release rates^[123]^.

The choice of loading methods varies significantly depending on the physicochemical properties of the drug. Small molecules, while easily permeable across membranes, carry a risk of leakage and are typically loaded using incubation, extrusion, or sonication methods^[124-127]^. For nucleic acid drugs, characterized by large molecular weight and dense negative charge, electroporation is the preferred approach^[128,129]^. The core challenge for protein drugs lies in preserving their three-dimensional conformation. While sonication and freeze-thaw methods avoid harsh chemical processing, they necessitate vigilance against aggregation-induced inactivation. In contrast, endogenous fusion expression, though time-consuming, maximizes preservation of protein activity^[117]^.

### Engineered EVs delivering small-molecule drugs overcome drug resistance

Engineered EVs delivering small-molecule chemotherapeutic drugs demonstrate unique advantages in overcoming MDR, substantially restoring drug sensitivity in resistant tumor cells. For instance, co-incubation of docetaxel with human umbilical cord mesenchymal stem cell-derived EVs (hUCMSC-Exos) significantly enhanced its cytotoxic effect against SKOV3 and the resistant SKOV3/DTX cell line, promoting apoptosis. This mechanism may involve EV-carried miR-146a regulating the phosphatidylinositol 3-kinase (PI3K)/Akt pathway to reduce resistance^[130]^. EVs from cisplatin-loaded M1 macrophages or NK cell-derived EVs more effectively induced apoptosis in both parental and drug-resistant ovarian cancer cells. Compared to free cisplatin, engineered EVs achieve stronger antitumor efficiency at lower concentrations by increasing intracellular drug accumulation, reducing efflux, and interfering with DNA repair^[131,132]^. EVs enter cells directly via membrane fusion or receptor-mediated endocytosis, effectively bypassing drug efflux pumps such as P-gp that are overexpressed on the cell membrane. This enhances intracellular drug concentration within tumor cells, thereby overcoming drug resistance.

Beyond bypassing efflux pumps, EV-based drug delivery systems hold promise for improving drug distribution in tumor tissues and reducing systemic toxicity due to their inherent targeting capabilities and biocompatibility. EVs loaded with curcumin demonstrated stronger anticancer activity against cervical cancer than free curcumin in both *in vitro* and *in vivo* studies, while exhibiting lower toxicity to normal cells^[133]^. This suggests that engineered EVs not only restore chemotherapy sensitivity in drug-resistant cells but also offer novel approaches to reducing systemic toxicity in the heart, liver, and kidneys by enhancing tumor targeting and minimizing exposure to normal tissues.

### Engineered EVs delivering nucleic acid drugs to reverse drug resistance

Engineered EVs serve as ideal carriers for nucleic acid-based drug delivery. Leveraging their inherent biocompatibility, low immunogenicity, and excellent transmembrane capabilities, they can precisely introduce small interfering RNA (siRNA), miRNA, and Clustered Regularly Interspaced Short Palindromic Repeats (CRISPR) systems into target cells, providing a strategy to reverse tumor drug resistance at the genetic level.

In reversing MDR using siRNA, EVs demonstrate highly efficient delivery and gene silencing capabilities. Delivery of nucleic acid drugs targeting P-gp via EVs effectively suppresses its overexpression, thereby reducing the efflux of chemotherapy drugs from tumor cells and restoring sensitivity^[134]^. Similarly, targeting other key drug resistance and survival-related genes, such as connective tissue growth factor or the N6-methyladenosine (m6A) demethylase fat mass and obesity-associated protein (FTO), via EV-mediated siRNA delivery not only achieves specific gene silencing but also effectively suppresses tumor growth or improves neurological function in disease models^[135,136]^. This demonstrates that delivering siSTAT3 (signal transducer and activator of transcription 3 siRNA), siHIF-1α (siRNA targeting Hypoxia-Inducible Factor 1-alpha), and other agents to interfere with anti-apoptotic signaling or tumor metabolism represents a highly promising research direction.

In terms of miRNA regulation, EV delivery of miRNA mimics or inhibitors can remodel complex RNA regulatory networks within tumors. Delivery of miR-497 inhibits angiogenesis-related genes in NSCLC^[137]^, whereas delivery of miR-122 or miR-199a enhances HCC cell sensitivity to DOX or reverses chemoresistance by targeting the mechanistic Target of Rapamycin (mTOR) pathway, respectively^[138,139]^. For oncogenic miRNAs such as miR-21, inhibitors delivered via EVs effectively upregulate tumor suppressor gene expression, enhance chemotherapy efficacy, and overcome resistance in glioblastoma and gastric cancer^[140,141]^. This demonstrates EVs’ potent capacity for precisely regulating drug-resistance-associated miRNA networks. Furthermore, EVs enable the co-delivery of nucleic acids and chemotherapeutic agents. Simultaneous delivery of 5-FU and miR-21 inhibitors downregulates miR-21, restores phosphatase and tensin homolog (PTEN) and human MutS homolog 2 (hMSH2) expression, and synergistically induces apoptosis and cell cycle arrest. This strategy demonstrates significant reversal of drug resistance both *in vitro* and *in vivo*^[128]^.

Furthermore, EVs are at the forefront of exploration as delivery vehicles for CRISPR/CRISPR-associated protein 9 (*Cas9*) gene editing systems. Using EVs derived from O‑type red blood cells (RBCs) to deliver CRISPR-Cas9 system components such as Cas9 mRNA has been shown to be feasible with low cytotoxicity^[142]^. This provides crucial early-stage technical exploration and proof-of-concept for developing EVs into gene editing tools capable of targeting and correcting or knocking out disease-causing genes, including drug-resistant mutations. Engineered EVs, by delivering siRNA, miRNA regulators, and CRISPR systems, enable multi-level precision interventions targeting drug-resistant genes and pathways, ranging from post-transcriptional silencing and epigenetic regulation to gene editing. This approach offers a multifunctional, high-potential nanotherapeutic platform for fundamentally reversing tumor MDR.

### EV-nanomaterial hybrid systems

EV-nanomaterial hybrid systems integrate natural EVs with engineered nanomaterials to establish novel delivery platforms that combine biological functionality with engineered advantages^[143,144]^. EV-liposome hybrids represent a typical example of such systems. They effectively preserve the bioactive components of the EV membrane, such as key transmembrane proteins CD47, CD55, and CD59. These proteins confer significant immune-evasion capabilities to the hybrids by transmitting “don’t eat me” signals or suppressing the complement system, thereby overcoming tumor resistance^[145-148]^. Simultaneously, these hybrids leverage the well-established drug delivery capabilities of liposomes, including high encapsulation efficiency and large internal aqueous phase space, to substantially enhance loading capacity for diverse therapeutic agents^[149]^. Studies indicate that such hybrids exhibit greater stability in plasma than either individual component alone. Furthermore, by leveraging the homing properties and tunable targeting capabilities of EVs, genetically engineered EVs expressing ligands or homing peptides fused to transmembrane proteins on their surface can achieve targeted delivery to cells expressing cognate receptors^[148-150]^.

**Figure 4:**
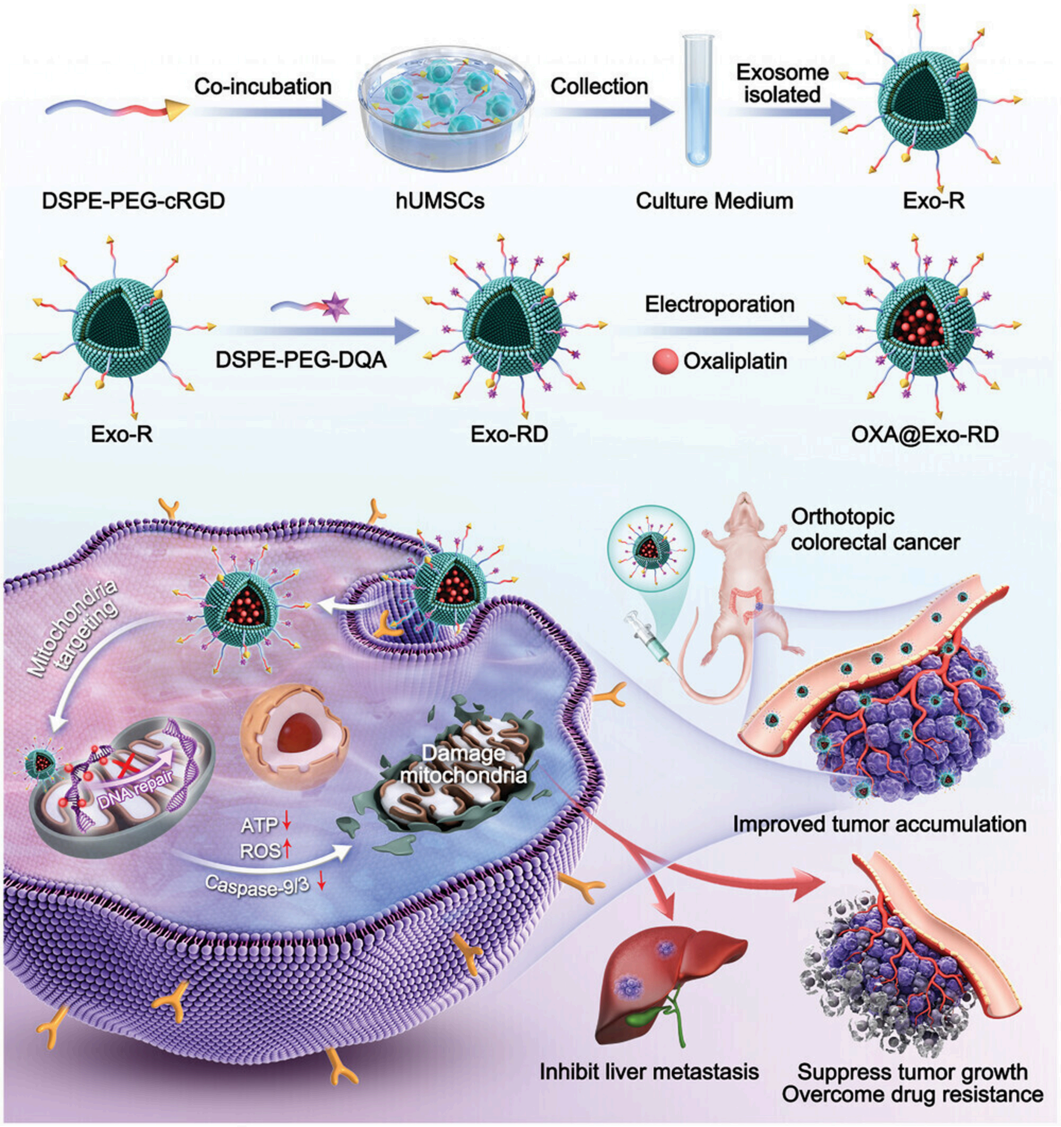
Schematic diagram for the construction of OXA@Exo-RD nanocomposite and their applications in reversing chemoresistance and anti-metastasis^[155]^. DSPE-PEG-cRGD: 1,2-distearoyl-sn-glycero-3-phosphoethanolamine-polyethylene glycol-cyclic arginylglycylaspartic acid; hUMSCs: human umbilical cord mesenchymal stem cells; Exo-R: cRGD-modified exosomes; DSPE-PEG-DQA: 1,2-distearoyl-sn-glycero-3-phosphoethanolamine-polyethylene glycol-dequalinium; Exo-RD: dequalinium-modified exosomes; OXA: oxaliplatin; OXA@Exo-RD: oxaliplatin-loaded dequalinium-modified exosomes; ATP: adenosine triphosphate; ROS: reactive oxygen species.

Beyond this, EV-nanoparticle hybrids represent another emerging class of nanomedicine delivery systems. These structures form a “core-shell” architecture by using EV membranes as a biomimetic coating to envelop synthetic nanoparticles such as poly(d,l-lactide-co-glycolide) (PLGA) polymer nanoparticles^[151]^, gold nanoparticles^[152,153]^, or magnetic Fe_3_O_4_ nanoparticles^[154]^. The biomimetic recognition and camouflage functions provided by the EV membrane not only prolong the blood-circulation half-life of nanoparticles but also promote carrier accumulation at the lesion site through their natural homing properties^[151,154]^. For instance, the research team developed a targeted mitochondrial functional nanomedicine (OXA@Exo-RD) that encapsulates OXA. The released OXA not only damaged mitochondrial DNA to exert antitumor effects but also prevented the initiation of DNA repair, thereby overcoming chemotherapy resistance [Figure 4]^[155]^. In addition, EV-magnetic nanoparticle hybrids loaded with DOX enable more precise tumor targeting under external magnetic fields^[154]^, while EV-coated PLGA nanoparticles achieve orders-of-magnitude enhancement in uptake efficiency by target cells^[151]^. The EV-nanomaterial hybrid system combines the biological properties of natural vesicles with the engineered advantages of synthetic materials. In reversing tumor MDR, its enhanced targeting, prolonged circulation time, and immune-evasion capabilities ensure that higher drug doses can be delivered to resistant tumor sites^[146,156,157]^.

### Engineered EVs modulate the immune microenvironment to overcome immune resistance

Immune checkpoint inhibitor (ICI) therapies frequently encounter resistance in clinical applications due to the suppressive nature of the tumor immune microenvironment (TME). As a novel delivery platform, EVs can precisely deliver specific immunomodulatory molecules to the TME, thereby overcoming ICI resistance^[158,159]^.

Tumor cells can exhaust T cells by releasing PD-L1-bearing EVs, thereby mediating immune escape^[160]^. To reverse this process, engineered EVs have been employed to deliver anti-PD-L1 molecules. Researchers have developed a dual-targeted drug delivery system by conjugating PD-L1 antibodies and CD40 antibodies to EV membranes. Upon reaching the tumor site, the PD-L1 antibodies are released and bind to PD-L1 on tumor cell surfaces, antagonizing its immunosuppressive function^[161]^. This approach enhances the therapeutic efficacy of ICI by restoring T cell function through decreasing PD-L1 expression on both tumor and immune cells.

Engineered EVs can also deliver agonists targeting the cyclic guanosine monophosphate adenosine monophosphate (GMP-AMP) synthase - stimulator of interferon genes (cGAS-STING) pathway or toll-like receptor (TLR) to activate the type I interferon pathway and remodel the immunosuppressive TME. One study engineered a multifunctional EV, Exo@MnIO&BG (exosome loaded with manganese-doped iron oxide nanoparticles, GW4869, and L-buthionine sulfoximine), which enhances the efficacy of cGAS-STING-based immunotherapy and improves the TME by promoting ferroptosis in tumor cells^[162]^. Furthermore, surface modification of EVs with TLR agonists can effectively induce antitumor immune responses and enhance tumor vaccine efficacy^[163]^. Another study utilized EVs loaded with TLR3 agonists and human neutrophil elastase (ELANE), which were surface-modified with targeting molecules. These engineered EVs can target DCs and activate conventional DCs *in situ*, thereby cross-activating cytotoxic T lymphocyte responses^[164]^. These approaches activate innate immune signaling pathways, promote DC maturation and antigen presentation, ultimately driving CD8⁺ T cell activation and tumor killing.

The TME comprises multiple immune cell components that collectively influence the outcome of immunotherapy. Engineered EVs offer the potential for broad immunomodulation of these cells. DC-derived EVs loaded with tumor antigens can effectively activate T and B lymphocyte immune responses, suppressing melanoma growth and delaying tumor recurrence^[165]^. EVs derived from NK cells release functional proteins such as perforin and granzyme, exhibiting cytotoxicity toward tumor cells while possessing smaller size and enhanced tissue permeability^[166,167]^. For immunosuppressive cells, studies have loaded galectin-9 (Gal-9) siRNA and OXA into bone marrow mesenchymal stem cell-derived EVs (MSC-EVs). By blocking the Gal-9/dectin-1 axis, this approach reverses the immunosuppressive effects of M2 tumor-associated macrophages while promoting DC maturation and cytotoxic lymphocyte infiltration^[168]^. More comprehensive strategies, such as genetically engineered EV αCD3-αEGFR-PD-1-OX40L GEMINI-Exos (genetically engineered multifunctional immune-modulating exosomes from Expi293F cells displaying PD-1, OX40L, anti-CD3, and anti-EGFR), achieve precise modulation of the immune microenvironment by fusing targeting antibodies with immunoregulatory proteins on the EV membrane. Simultaneously displaying antibodies targeting CD3 and EGFR on its surface while co-expressing PD-1 and OX40L, this platform redirects and activates T cells to kill EGFR-positive TNBC cells, significantly inhibiting tumor growth in mouse models^[169]^. These studies demonstrate that through careful design, engineered EVs can serve as a multifunctional platform to simultaneously target and regulate diverse immune cells within the TME, including DCs, NK cells, Tregs, and MDSCs, coordinating innate and adaptive immune responses to systematically overcome immune resistance.

Engineered EVs can effectively remodel the suppressive TME by precisely delivering immunomodulatory molecules such as siPD-L1 (small interfering RNA targeting PD-L1), STING/TLR agonists, or by presenting multifunctional ligands. This enhances antitumor immune responses and offers a solution for overcoming current ICI treatment resistance.

### Engineered EVs synergize with natural products/TCM active components to reverse drug resistance

The mechanisms underlying tumor MDR are complex, involving multilevel factors such as multiple signaling pathways, metabolic reprogramming, and immune microenvironment imbalance. Traditional single-target drugs struggle to effectively address these challenges. TCM and its active components, with their multicomponent, multipathway, and multitarget advantages, align well with this complex challenge, emerging as crucial tools for reversing drug resistance^[170]^. However, most natural active components suffer from poor water solubility, low bioavailability, and nonspecific distribution, limiting their clinical translation. Engineered EVs offer an innovative solution to this challenge through their superior biocompatibility, low immunogenicity, and inherent targeted delivery capabilities. By integrating the multipathway regulatory advantages of natural products with the precise delivery properties of EVs, a triple-regulation system of “drug-carrier-microenvironment” is established, providing a novel strategy for overcoming tumor drug resistance.

Multiple active components in TCM can exert key roles by directly regulating drug resistance-related signaling pathways. For instance, Tripteryginine loaded into EVs has been demonstrated to inhibit nuclear factor kappa B (NF-κB) activation induced by tumor necrosis factor-α and activate apoptosis through the endoplasmic reticulum stress pathway, thereby suppressing the proliferation and metastasis of NSCLC^[171]^. Certain natural products can also indirectly influence tumor drug resistance phenotypes by regulating EV-mediated intercellular communication networks. For instance, EV-mediated antitumor effects of corydalis glycoside occur by promoting the release of miR-7-5p-containing EVs from glioblastoma cells, thereby inhibiting the EGFR/PI3K/Akt signaling pathway^[172]^. β-Elemene-containing EVs reverse MDR by upregulating miR-34a and PTEN expression in breast cancer-resistant cells while downregulating resistance-associated miR-452 and P-gp expression^[173]^. Additionally, components such as epigallocatechin gallate (EGCG) and curcumin can influence the polarization state of tumor-associated macrophages by modulating the composition of specific miRNAs within EVs, thereby improving the immunosuppressive microenvironment^[174,175]^. Engineered EVs, serving as highly efficient delivery vehicles for natural products, can significantly overcome their pharmacokinetic limitations, enhancing therapeutic efficacy and targeting. For instance, EVs derived from macrophages loaded with PTX exhibited more than a 50-fold increase in cytotoxicity against P-gp-positive drug-resistant cells, demonstrating potent potential for reversing drug resistance^[126]^. Further research combined self-assembled nanomicelles formed from tanshinone IIA and glycyrrhizic acid with serum-derived EVs, with the immune agonist cytidine-phosphate-guanosine (CpG) anchored to their surface. This successfully established a smart EV system integrating targeted delivery and immune modulation capabilities^[176]^.

Natural products and active components of TCM reverse drug resistance at the molecular and microenvironmental levels by multitargeted intervention in resistance-related pathways and regulation of EV-mediated miRNA networks and cellular metabolism. Engineered EVs, serving as intelligent delivery platforms, effectively address inherent limitations of natural products and enable precise targeting and synergistic enhancement through surface modification. The integrated approach combining “multi-target regulation-precision delivery-microenvironment remodeling” holds promise for overcoming resistance barriers in cancer therapy.

### Safety, production standards, and scalability of engineered EVs

Before engineered EVs can advance to clinical applications, safety assessment, standardized production, and quality control represent critical hurdles that must be overcome. Preliminary safety studies on EVs from various sources have been initiated. Injecting mesenchymal stem cell-derived EVs (MSC-Exos) into patients after craniotomy showed no adverse events such as hematoma, edema, severe meningitis, or brain abscess at the injection site. No significant changes in blood cell counts or liver/kidney function were observed in the short term^[177]^. A Phase I clinical trial for anal fistula treatment also reported no significant adverse reactions after one year of follow-up^[178]^. Preliminary toxicological evaluations, including skin sensitization, acute oral toxicity, phototoxicity, eye irritation, and skin irritation, revealed no evident toxic reactions^[179]^. However, these studies typically featured short follow-up periods and limited sample sizes, which are insufficient to comprehensively assess long-term safety. Additionally, the potential long-term epigenetic effects of bioactive substances carried by MSC-Exo represent a novel challenge that is difficult to evaluate using traditional toxicological models.

In terms of production standards and quality control, achieving large-scale production compliant with Good Manufacturing Practice (GMP) requirements remains a primary bottleneck. Current challenges include difficulties in precisely controlling actual yields, significant batch-to-batch quality variation, and the absence of standardized extraction and purification processes. Future development will involve establishing a comprehensive quality assurance system across the entire supply chain. This includes utilizing serum-free media with clearly defined chemical compositions at the source to minimize batch-to-batch variability^[180]^, and implementing internationally recognized gold standards for efficacy evaluation based on functional indicators at the endpoint^[181]^. Only by overcoming challenges in standardized production, batch consistency, and stringent quality control can engineered EVs meet regulatory requirements and achieve reliable clinical translation.

Among engineered EV approaches to reversing drug resistance, the EV-liposome hybrid system integrates the immune evasion capabilities of natural EVs with the high loading efficiency of liposomes. It demonstrates superior efficacy compared with single-carrier systems across multiple drug-resistant models. Its production relies on relatively mature liposome preparation techniques, offering greater potential for large-scale manufacturing. In addition, utilizing EVs to deliver nucleic acid therapeutics targeting drug-resistant genes offers distinct advantages: a well-defined pathway and quantifiable endpoints that facilitate regulatory assessment. By directly silencing or editing genes driving resistance, this approach restores tumor cell sensitivity at its root cause. Substantial preclinical data have already been accumulated regarding siRNA delivery via this method. Finally, the combined strategy of engineered EVs modulating the TME can systematically overcome ICI resistance by simultaneously lifting immunosuppression and activating innate immunity. This multi-target synergistic approach aligns with the complexity of the tumor microenvironment and holds promise for generating stronger antitumor immune responses. In addition to the strategies discussed above, antibodies and antibody-drug conjugates (ADCs) also represent important classes of anticancer therapeutics. Studies have shown that EVs can mediate resistance to anticancer monoclonal antibody therapy. Aung *et al*. demonstrated that in aggressive B-cell lymphoma, tumor cells release exosomes carrying CD20 antigen, which bind to therapeutic anti-CD20 antibodies and consume complement, thereby protecting tumor cells from antibody-mediated complement-dependent cytotoxicity through a decoy effect^[182]^. Furthermore, EVs can also influence the efficacy of ADCs. Barok *et al*. noted that the target antigens of ADCs are widely expressed on the surface of EVs^[183]^. These vesicles can deliver ADCs to tumor cells that do not express the target antigen via a bystander effect, thereby enhancing antitumor efficacy. Concurrently, EVs can also contribute to ADC resistance through mechanisms such as decoy effects that consume ADCs, drug efflux via vesicle secretion, and transfer of ABC transporters. In summary, promising engineered EV formulations will likely evolve toward multimodal, dual-target approaches that simultaneously enhance tumor penetration and reshape the immune microenvironment, thereby synergistically tackling the complex challenge of MDR [Table 2].

**Table 2:** Research on engineered EVs reversing tumor drug resistance

**EV source**	**Engineered loading method**	**Loaded drug/molecule**	**Reversed drug resistance type**	**Treated tumor type**	**Ref.**
HEK293T cell-derived EVs	Incubation	exoASO-STAT6	Immunotherapy resistance	Colorectal cancer, hepatocellular carcinoma	[184]
Macrophage-derived EVs	Sonication	Paclitaxel	Pgp-mediated multidrug resistance	Drug-resistant lung cancer	[126]
Macrophage-derived EVs	Endogenous loading	miR-365 antagonist	Gemcitabine resistance	Pancreatic ductal adenocarcinoma	[185]
AMSC-derived EVs	Endogenous loading	miRNA-122	Chemosensitization	Liver cancer	[138]
AMSC-derived EVs	Endogenous loading	miRNA-199a	Chemosensitization	Liver cancer	[138]
MSC-derived EVs	Endogenous loading	Anti-miRNA-9-Cy5	Temozolomide resistance	Glioblastoma	[186]
HEK293T cell-derived EVs	Endogenous loading	BCR-ABL siRNA/Imatinib	Reversal of imatinib resistance	Chronic myeloid leukemia	[187]
hUCMSC-derived EVs	Endogenous loading	miR-146a	Docetaxel resistance, taxane resistance	Ovarian cancer	[130]
Milk-derived EVs	Ultracentrifugation	Anthocyanin	Cisplatin resistance	Ovarian cancer	[134]
Umbilical cord blood M1 macrophage-derived EVs	Sonication	Cisplatin	Cisplatin resistance	Ovarian cancer	[131]
NK cell-derived EVs	Electroporation	Cisplatin	Cisplatin resistance	Ovarian cancer	[132]
HEK293T cell-derived EVs	Electroporation	5-fluorouracil & miR-21 inhibitor	5-fluorouracil resistance	Colorectal cancer	[128]
HEK293T cell-derived EVs	Endogenous loading/electroporation	Oxaliplatin & lncRNA PGM5-AS1	Oxaliplatin resistance	Metastatic colorectal cancer/Colon cancer	[188]
HEK293T cell-derived EVs	Endogenous loading/electroporation	FAK-targeting siRNA	Cetuximab resistance	Colorectal cancer	[158]
hUMSC-derived EVs	Surface engineering/electroporation	Oxaliplatin & mitochondrial-targeting molecule DQA	Oxaliplatin resistance	Colorectal cancer	[155]
Homologous glioma cell (GL261)-derived EVs	Sonication/incubation	Temozolomide & dihydrotanshinone	Temozolomide resistance	Glioblastoma	[189]
Human ovarian cancer cell (A2780)-derived EVs	Ultracentrifugation	Tetramethylpyrazine	Multidrug resistance (e.g., PTX resistance)	Ovarian cancer	[190]
Human gastric cancer cell (SGC-7901/MGC-803)-derived EVs	Endogenous loading	microRNA-107	5-FU resistance, Cisplatin resistance (cross-resistance)	Gastric cancer	[191]

exoASO-STAT6: Antisense oligonucleotide targeting signal transducer and activator of transcription 6; EVs: extracellular vesicles; HEK293T: human embryonic kidney 293T cells; Pgp: P-glycoprotein; miR: microRNA; AMSC: amniotic mesenchymal stem cell; MSC: mesenchymal stem cell; hUCMSC: human umbilical cord mesenchymal stem cell; NK: natural killer; siRNA: small interfering RNA; lncRNA: long non-coding RNA; FAK: focal adhesion kinase; hUMSC: human umbilical cord mesenchymal stem cell; GL261: murine glioma cell line; A2780: human ovarian cancer cell line; SGC-7901: human gastric cancer cell line; MGC-803: human gastric cancer cell line; PTX: paclitaxel; 5-FU: 5-fluorouracil; DQA: mitochondrial-targeting molecule dodecyl triphenylphosphonium cation derivative.

### Bottlenecks and solutions for the clinical translation of engineered EVs

Despite the rapid advancement of engineered EVs as drug delivery platforms, their translation from bench to bedside remains constrained by multiple practical and regulatory bottlenecks^[192]^.

One of the foremost challenges lies in achieving scalable and reproducible manufacturing. Current production strategies for engineered EVs often rely on cell culture systems that exhibit batch-to-batch variability, low yields, and limited scalability. In addition, differences in donor cell types, culture conditions, and engineering approaches can significantly influence EV composition and functional performance. To overcome these limitations, the development of standardized producer cell lines, bioreactor-based large-scale production systems, and well-defined upstream and downstream processing workflows will be essential. Emerging approaches such as EV mimetics and cell-free synthetic vesicles may further improve scalability and controllability^[193]^.

Cargo-loading efficiency and stability also remain critical barriers. Both endogenous loading (via donor cell engineering) and exogenous loading (e.g., electroporation, sonication, chemical transfection) are associated with variable efficiency, potential cargo degradation, and unintended alterations in vesicle integrity. Moreover, maintaining cargo stability during storage and systemic circulation is a significant challenge^[194]^. Advances in loading technologies, including membrane-fusion strategies, genetically encoded loading systems, and stimuli-responsive encapsulation methods, are being explored to enhance loading precision and stability.

Another major concern is target specificity and *in vivo* biodistribution. Engineered EVs often exhibit preferential accumulation in organs such as the liver, spleen, and lungs due to clearance by the mononuclear phagocyte system, which limits their effective delivery to tumor sites. Furthermore, off-target uptake may lead to reduced therapeutic efficacy and potential adverse effects^[195]^. Surface-engineering strategies, including ligand modification, antibody conjugation, and genetic display of targeting moieties, are being actively investigated to improve tissue specificity and cellular uptake.

Safety, immunogenicity, and quality control represent additional critical considerations for clinical translation. Although EVs are generally considered biocompatible, their biological origin raises concerns regarding potential immunogenicity, horizontal transfer of oncogenic material, and long-term safety. Rigorous characterization of EV composition, purity, and biological activity, along with the establishment of stringent quality control criteria, will be required. The implementation of GMP-compliant production and standardized release criteria will be indispensable for clinical application.

Finally, regulatory and translational hurdles further complicate clinical implementation. Engineered EVs occupy a complex regulatory space at the interface of biologics, drug delivery systems, and cell-derived therapeutics, resulting in unclear classification and approval pathways in many jurisdictions. In addition, there is a lack of large-scale, well-controlled clinical trials demonstrating their safety and efficacy. Addressing these challenges will require close collaboration among researchers, clinicians, industry stakeholders, and regulatory agencies to establish clear guidelines, harmonized standards, and robust clinical evidence.

In summary, bridging the gap between laboratory innovation and clinical application will depend on advances in scalable manufacturing, precise engineering technologies, targeted delivery strategies, and regulatory standardization. Continued interdisciplinary efforts will be essential to unlock the full therapeutic potential of engineered EVs in oncology.

## PRECLINICAL AND CLINICAL TRANSLATION

Despite numerous studies demonstrating the potential of EVs to reverse tumor drug resistance, their ultimate clinical value hinges on successfully bridging the gap from laboratory research to large-scale, safe, and rigorously controlled clinical translation. This section will focus on the core bottlenecks encountered by current EV therapeutics, particularly engineered products targeting resistance reversal, during clinical translation. Based on existing preclinical and clinical explorations, it will also outline promising future directions for breakthroughs.

### Evidence of engineered evs reversing drug resistance in animal models

As a novel delivery platform, EVs have demonstrated the capacity to overcome resistance to chemotherapy, targeted therapy, and immunotherapy in diverse preclinical tumor models. Notably, the majority of successful preclinical studies share a common feature: engineered EVs are used to intervene in dominant resistance drivers rather than secondary adaptive pathways.

In the context of targeted therapy resistance, engineered EVs have been employed to deliver nucleic acid therapeutics that directly suppress resistance-associated oncogenic signals. For example, in a pancreatic cancer model, EV-mediated delivery of plasmids encoding Cas9 protein and single guide RNA (sgRNA) targeting mutant Kras^G12D^ effectively inhibited tumor growth both *in vivo* and *in vitro*, providing proof-of-concept evidence that engineered EVs can overcome mutation-driven targeted resistance^[196]^. Similarly, in an NSCLC model, EV-based co-delivery of an EGFR aptamer (EGFRapt) and survivin siRNA (siSurvivin) in combination with cisplatin demonstrated enhanced antitumor efficacy in animal studies^[197]^. These studies highlight the translational feasibility of using engineered EVs to precisely modulate genetically defined resistance mechanisms.

In reversing immunotherapy resistance and remodeling the immunosuppressive tumor microenvironment, multiple engineered EV strategies have demonstrated robust efficacy. EV-derived antisense oligonucleotides (exoASO-STAT6), engineered to express interleukin (IL)-3 on their surface and loaded with STAT6 antisense oligonucleotides (ASO), successfully reprogrammed M2 tumor-associated macrophages into proinflammatory M1 phenotypes. In mouse models of CRC and HCC, both intratumoral and intravenous administration resulted in dose-dependent tumor growth inhibition and superior efficacy compared with free ASO^[184]^. These findings support the concept that immune resistance represents a highly tractable target for engineered EV-based intervention.

Addressing resistance mediated by immune checkpoint molecules, studies have demonstrated that inhibiting PD-L1 secretion via tumor-derived EVs is critical for restoring antitumor immunity. In mouse models, a biomimetic EV (apoA1-bExo) designed to block EV PD-L1 secretion effectively eradicated implanted tumors and significantly enhanced the therapeutic efficacy of anti-PD-L1 antibodies^[198]^. Concurrently, engineered EVs loaded with neoantigens and functioning as nanovaccines induced strong proinflammatory cytokine responses in melanoma and CRC models, promoting CD4⁺ and CD8⁺ T cell proliferation and improving immune homeostasis^[165]^. In HCC models, α-fetoprotein (AFP)-enriched dendritic cell-derived EVs (Dex) elicited potent antigen-specific immune responses, characterized by increased intratumoral CD8⁺ T cells and IFN-γ levels, reduced regulatory T cells, and diminished immunosuppressive cytokines^[199]^. Similar immune remodeling effects were observed when Dex therapy was combined with microwave ablation in liver cancer models^[200]^. Collectively, these studies underscore the strong translational rationale for engineered EVs in overcoming immunotherapy resistance.

The route of administration represents another critical determinant of translational success. Following intravenous injection, MSC-Exos predominantly accumulate in the liver, spleen, and lungs^[201,202]^. In contrast, arterial administration circumvents pulmonary first-pass sequestration, enabling broader organ distribution, including the heart and kidneys^[203]^. For localized or organ-specific applications, intratumoral injection^[184]^, intranasal administration^[204]^, and nebulized inhalation^[205]^ offer enhanced local bioavailability and improved targeting precision. Moreover, EVs exhibit an intrinsic tendency to home toward pathological microenvironments, such as sites of acute kidney injury^[206]^. These observations highlight the necessity of tailoring administration routes to therapeutic objectives, disease context, and safety considerations in future clinical translation.

In summary, preclinical evidence demonstrates that engineered EVs can effectively reverse resistance to targeted therapy and immunotherapy by directly intervening in dominant resistance pathways, reprogramming immune cell function, and reshaping the tumor microenvironment. However, successful clinical translation will depend not only on efficacy but also on scalable manufacturing, reproducible cargo loading, controlled biodistribution, and rigorous safety evaluation. Together, these studies provide a rational framework for prioritizing engineered EV strategies with the highest likelihood of clinical impact.

### Clinical evidence of EV liquid biopsy in drug resistance monitoring

Timely identification of drug resistance during cancer treatment is critical for optimizing therapeutic strategies and improving patient outcomes. As a rapid, noninvasive, and repeatable approach for dynamic monitoring, EV-based liquid biopsy has emerged as a promising tool for predicting and evaluating resistance to chemotherapy, targeted therapy, and immunotherapy. Importantly, its clinical value lies not in single time-point diagnosis, but in longitudinal surveillance and early warning of therapeutic failure.

Specific ncRNAs carried by EVs have been reported as potential biomarkers for resistance prediction. In patients with NSCLC, altered expression of plasma EV-derived miR-184 and miR-3913-5p has been associated with osimertinib resistance^[207]^, while EV circRNA-102481 has been proposed as a diagnostic marker for EGFR-TKI resistance ^[208]^. In breast cancer, elevated EV lncRNA HOTAIR (homeobox transcript antisense intergenic RNA) correlates with poor response to neoadjuvant chemotherapy and tamoxifen treatment^[209]^. In ovarian cancer, increased EV miR-675-3p and miR-429 contribute to cisplatin resistance via activation of PI3K/Akt pathway, with miR-675-3p levels positively correlating with cisplatin half maximal inhibitory concentration (IC_50_) values^[210]^. Collectively, these findings support the feasibility of EV ncRNAs as resistance-associated biomarkers, although current evidence remains largely correlative and retrospective in nature.

Among EV biomarkers, EV PD-L1 (exoPD-L1) demonstrates particularly strong clinical relevance in the context of ICI therapy^[211]^. Baseline exoPD-L1 levels correlate with treatment response in NSCLC patients, while dynamic changes during therapy provide real-time insight into resistance evolution, decreasing during effective treatment and increasing upon disease progression or recurrence^[212,213]^. Predictive models integrating exoPD-L1 with immune-related markers such as CD28 further improve prognostic accuracy^[214]^. These findings demonstrate that exoPD-L1 serves not only as a baseline predictive biomarker but also provides real-time insights for evaluating ICI treatment efficacy and detecting resistance through noninvasive, repeatable dynamic monitoring. Compared with conventional monitoring approaches, EV-based liquid biopsy offers advantages in noninvasiveness, repeatability, and molecular stability due to lipid bilayer protection^[215]^. However, limitations remain, including the lack of standardized isolation protocols, insufficient prospective validation, and relatively high costs^[216]^. At present, EV liquid biopsy should be regarded as a complementary tool rather than a replacement for imaging or conventional serum markers, with continued standardization expected to enhance its role in resistance monitoring and personalized treatment adjustment^[217]^.

### Current status of clinical trials for EV therapeutics and their potential for “reversing drug resistance”

Although EVs have demonstrated strong potential for reversing tumor drug resistance in preclinical studies, their clinical application remains at an early exploratory stage. To date, no registered clinical trial has defined “reversal of drug resistance” as a primary efficacy endpoint. Existing trials mainly focus on evaluating the safety, feasibility, and preliminary biological activity of EVs as natural delivery vehicles or immunomodulators, thereby laying the groundwork for future development of engineered EV platforms targeting resistance reversal^[218]^.

Several early-phase clinical trials, particularly those involving MSC-EVs and Dex, have demonstrated favorable safety and tolerability profiles across oncological and non-oncological indications. In cancer immunotherapy, Dex-based vaccines showed acceptable safety in Phase I trials for advanced NSCLC and metastatic melanoma, with evidence of peripheral immune activation, including enhanced NK cell activity^[218,219]^. In addition, a Phase I clinical study (NCT01294072) evaluated plant-derived EV-like nanoparticles loaded with curcumin in patients with colon cancer, with endpoints including safety, tolerability, and biological activity. Beyond oncology, MSC-EV trials in diseases such as graft-versus-host disease, coronavirus disease 2019 (COVID-19), and osteoarthritis further support the clinical tolerability of EV-based therapies. Importantly, these trials predominantly enrolled patients with advanced or treatment-refractory disease, closely matching the anticipated target population for future EV-based strategies aimed at overcoming established drug resistance^[220,221]^.

Despite these advances, a clear translational gap remains. Current clinical studies have not been designed to directly assess resistance reversal to chemotherapy, targeted therapy, or immunotherapy. Future trials should therefore prioritize well-defined patient cohorts with documented therapeutic resistance and incorporate resistance-focused endpoints, such as dynamic changes in resistance-associated biomarkers, restoration of treatment sensitivity, durable disease control after resistance onset, and prolongation of progression-free survival. By leveraging accumulated clinical experience in safety, dosing, administration routes, and biomarker integration, the rational translation of engineered EV-based therapeutics specifically targeting drug resistance can be significantly accelerated.

### Key bottlenecks in clinical translation and future strategies

The clinical translation of engineered EVs is currently constrained by three major bottlenecks: manufacturing standardization, safety evaluation, and regulatory uncertainty. At the manufacturing level, the lack of unified standards remains a central challenge. Variations in parental cell sources (e.g., MSC *vs*. tumor cells), culture conditions (serum-containing *vs*. serum-free systems), and isolation methods (density gradient centrifugation, ultrafiltration, or SEC) can markedly affect EV yield, physicochemical properties, and therapeutic performance^[222-225]^. Even minor process deviations may result in substantial batch-to-batch heterogeneity, complicating quality control and reproducibility^[226]^. In addition, standardized methodologies for defining critical quality attributes, such as particle size distribution, cargo loading efficiency, and functional potency, are still lacking^[227]^. Storage and preservation further limit translation, as conventional conditions may compromise stability and bioactivity^[228,229]^, while alternative approaches such as lyophilization require further validation^[230]^.

Safety considerations represent a second critical bottleneck. Although EVs are often regarded as biocompatible nanocarriers, their complex biological composition introduces potential risks, including immune activation, cytokine release, and off-target effects^[231]^. According to the position of the International Society for Extracellular Vesicles, when therapeutic efficacy is attributed primarily to the loaded drug rather than the vesicle itself, EVs may be classified as excipients requiring safety characterization. However, when EVs exert intrinsic biological activity, comprehensive mechanistic and toxicological evaluation becomes mandatory for early-phase clinical trials^[194]^. At present, the absence of harmonized safety testing frameworks and the lack of clear regulatory guidance substantially increase uncertainty in clinical development of engineered EV therapeutics.

Collectively, these challenges reflect the absence of an integrated translational framework spanning manufacturing, characterization, storage, safety assessment, and regulatory alignment. Future progress will require the establishment of GMP-compliant and scalable production pipelines, prioritization of safer and more controllable parental cell sources such as MSC, and development of robust, cost-effective storage solutions suitable for clinical deployment. Equally important is coordinated collaboration among academia, industry, and regulatory agencies to define unified standards for quality control, safety evaluation, and clinical trial design. Incorporation of clinically relevant endpoints, particularly biomarkers indicative of resistance reversal, will be essential for advancing engineered EV-based anti-drug resistance strategies from experimental platforms to practical clinical applications.

## CONCLUSION AND OUTLOOK

EVs play a pivotal dual-edged role in tumor drug resistance. On one hand, tumor cells utilize EVs as key messengers to transmit functional substances, including specific ncRNAs, proteins, and metabolites, thereby disseminating drug-resistant phenotypes to sensitive cells and immune cells within the tumor microenvironment. This drives a series of resistance mechanisms such as drug efflux, evasion of apoptosis, EMT, immune suppression, and metabolic reprogramming. On the other hand, engineered EVs offer a precise delivery platform for reversing resistance due to their excellent biocompatibility, low immunogenicity, and inherent targeting capabilities. Engineered EVs can efficiently deliver small-molecule chemotherapeutic agents, siRNA/miRNA targeting drug-resistant genes, CRISPR editing systems, and active components of TCM through endogenous or exogenous loading. They effectively bypass drug efflux pumps such as P-gp or intervene in drug resistance pathways at the genetic level, demonstrating their ability to reverse chemotherapy resistance, target resistance mechanisms, and overcome immune tolerance in multiple animal models. Among these, nucleic acid-based drug delivery methods demonstrate high clinical translation potential due to their precise targeting, while hybrid systems combining liposomes offer both biomimetic and engineered advantages.

However, the current major bottleneck in translation lies in the absence of a standardized system spanning the entire production, characterization, and storage pipeline. Consistency and comparability between batches are difficult to guarantee, while the long-term safety and well-defined regulatory pathway for engineered EVs still require systematic investigation. Although EV-based liquid biopsy offers a non-invasive tool for dynamic monitoring of drug resistance, its isolation and detection technologies also urgently need standardization.

EVs hold great promise in precision anti-drug resistance and personalized immunotherapy. Future research should focus on the following directions: First, utilize multi-omics technologies to deeply analyze the molecular landscape of EVs in the dynamic evolution of drug resistance, combined with artificial intelligence (AI)-assisted screening for novel predictive biomarkers. Second, promote the establishment of GMP-compliant, scalable production processes and unified quality control standards to lay the foundation for clinical translation. Finally, therapeutic strategies should advance toward “multimodal, dual-target” combination therapies. For instance, integrating engineered EVs with ICIs and natural products possessing multipathway regulatory advantages could establish an integrated treatment strategy encompassing “precision delivery-immune reprogramming-microenvironment regulation”. Through close collaboration among academia, industry, and regulatory bodies, engineered EVs hold promise to evolve from a highly promising research tool into a clinical weapon capable of overcoming tumor drug resistance challenges.

## DECLARATIONS

### Copyright

© The Author(s) 2026.
